# AgingReG: a curated database of aging regulatory relationships in humans

**DOI:** 10.1093/database/baad064

**Published:** 2023-10-06

**Authors:** Minghui Piao, Ke Feng, Xinyu Liu, Xuefeng Bai, Yuqi Zheng, Meiling Sun, Peng Zhao, Yani Wang, Xiaofang Ban, Jie Xiong, Chengyu Shi, Li Meng, Yuxin Liu, Li Yu, Jing Li, Shan Zhong, Xinjian Jiang, Yu Chen, Xin Sun, Yan Zheng, Jinwei Tian

**Affiliations:** Department of Cardiology, The Second Affiliated Hospital of Harbin Medical University, No. 246 Xuefu Road, Nangang District, Harbin 150086, China; The Key Laboratory of Myocardial Ischemia, Harbin Medical University, Ministry of Education, No. 246 Xuefu Road, Nangang District, Harbin 150086, China; College of Bioinformatics Science and Technology, Harbin Medical University, No. 157 Baojian Road, Nangang District, Harbin 150086, China; School of Medical Informatics, Daqing Campus, Harbin Medical University, No. 39 Xinyang Road, High Tech Zone, Daqing 163319, China; School of Medical Informatics, Daqing Campus, Harbin Medical University, No. 39 Xinyang Road, High Tech Zone, Daqing 163319, China; State Key Laboratory of Genetic Engineering, Human Phenome Institute and School of Life Sciences, Fudan University, No. 2005 Songhu Road, Yangpu District, Shanghai 200438, China; Department of Cardiology, The Second Affiliated Hospital of Harbin Medical University, No. 246 Xuefu Road, Nangang District, Harbin 150086, China; Department of Cardiology, The Second Affiliated Hospital of Harbin Medical University, No. 246 Xuefu Road, Nangang District, Harbin 150086, China; Department of Cardiology, The Second Affiliated Hospital of Harbin Medical University, No. 246 Xuefu Road, Nangang District, Harbin 150086, China; Department of Cardiology, The Second Affiliated Hospital of Harbin Medical University, No. 246 Xuefu Road, Nangang District, Harbin 150086, China; Department of Cardiology, The Second Affiliated Hospital of Harbin Medical University, No. 246 Xuefu Road, Nangang District, Harbin 150086, China; Department of Cardiology, The Second Affiliated Hospital of Harbin Medical University, No. 246 Xuefu Road, Nangang District, Harbin 150086, China; Department of Cardiology, The Second Affiliated Hospital of Harbin Medical University, No. 246 Xuefu Road, Nangang District, Harbin 150086, China; Department of Cardiology, The Second Affiliated Hospital of Harbin Medical University, No. 246 Xuefu Road, Nangang District, Harbin 150086, China; Department of Cardiology, The Second Affiliated Hospital of Harbin Medical University, No. 246 Xuefu Road, Nangang District, Harbin 150086, China; Department of Cardiology, The Second Affiliated Hospital of Harbin Medical University, No. 246 Xuefu Road, Nangang District, Harbin 150086, China; Department of Cardiology, The Second Affiliated Hospital of Harbin Medical University, No. 246 Xuefu Road, Nangang District, Harbin 150086, China; Department of Cardiology, The Second Affiliated Hospital of Harbin Medical University, No. 246 Xuefu Road, Nangang District, Harbin 150086, China; Department of Cardiology, The Second Affiliated Hospital of Harbin Medical University, No. 246 Xuefu Road, Nangang District, Harbin 150086, China; Department of Cardiology, The Second Affiliated Hospital of Harbin Medical University, No. 246 Xuefu Road, Nangang District, Harbin 150086, China; Department of Cardiology, Shenzhen People’s Hospital (The Second Clinical Medical College, Jinan University; The First Affiliated Hospital, Southern University of Science and Technology), No. 1017 Dongmen North Road, Luohu District, Shenzhen 518000, China; State Key Laboratory of Genetic Engineering, Human Phenome Institute and School of Life Sciences, Fudan University, No. 2005 Songhu Road, Yangpu District, Shanghai 200438, China; Department of Cardiology, The Second Affiliated Hospital of Harbin Medical University, No. 246 Xuefu Road, Nangang District, Harbin 150086, China; The Key Laboratory of Myocardial Ischemia, Harbin Medical University, Ministry of Education, No. 246 Xuefu Road, Nangang District, Harbin 150086, China; Key Laboratory of Emergency and Trauma of Ministry of Education, Hainan Medical University, No. 3 Xueyuan Road, Longhua District, Haikou 571199, China

## Abstract

Aging and cellular senescence are characterized by a progressive loss of physiological integrity, which could be triggered by aging factors such as physiological, pathological and external factors. Numerous studies have shown that gene regulatory events play crucial roles in aging, increasing the need for a comprehensive repository of regulatory relationships during aging. Here, we established a manually curated database of aging factors (AgingReG, https://bio.liclab.net/Aging-ReG/), focusing on the regulatory relationships during aging with experimental evidence in humans. By curating thousands of published literature, 2157 aging factor entries (1345 aging gene entries, 804 external factor entries and eight aging-related pathway entries) and related regulatory information were manually curated. The regulatory relationships were classified into four types according to their functions: (i) upregulation, which indicates that aging factors upregulate the expression of target genes during aging; (ii) downregulation, which indicates that aging factors downregulate the expression of target genes during aging; (iii) activation, which indicates that aging factors influence the activity of target genes during aging and (iv) inhibition, which indicates that aging factors inhibit the activation of target molecule activity, leading to declined or lost target activity. AgingReG involves 651 upregulating pairs, 632 downregulating pairs, 330 activation-regulating pairs and 34 inhibition-regulating pairs, covering 195 disease types and more than 800 kinds of cells and tissues from 1784 published literature studies. AgingReG provides a user-friendly interface to query, browse and visualize detailed information about the regulatory relationships during aging. We believe that AgingReG will serve as a valuable resource database in the field of aging research.

**Database URL:** https://bio.liclab.net/Aging-ReG/

## Introduction

Aging is a complex process involving multiple factors at molecular, cellular and tissue levels. Regulation of gene expression during aging plays a crucial role in specific cells and tissues, which leads to a variety of aging phenotypes. In recent years, the research and development of anti-aging drugs based on the molecular regulation mechanism of aging have become a hot topic in the field of aging. However, discovering effective aging factors and clarifying the regulatory processes are significant scientific challenges. Experimental evidence is the gold standard for identifying aging molecules. Previous studies have shown that some aging factors regulate the aging process by regulating the expression of target genes. For example, gastrokine 1 downregulates the expression of c-myc by inhibiting the binding of C-MYC to telomeric repeat binding factor 1 and accelerates the rate of cell aging (1). The complex gene regulatory relationships during aging have been verified using a large number of low-throughput biological experiments, but the experimental evidence is widely scattered in many literature studies and databases.

Several databases have been developed, including the well-known Aging Atlas (2), CSGene (3), DrugAge (4) and the Human Ageing Genomic Resources (5). These databases have provided valuable resources for aging research but do not deeply explore the gene regulatory relationships during aging. Therefore, it is highly desirable to construct a comprehensive resource of manually curated aging gene regulatory relationships, which provides comprehensive experimental evidence.

To fill this gap, we established AgingReG, a database of aging factors focusing on the regulatory relationships during aging with experimental evidence in humans. Through reviewing 1784 articles, we have manually classified the regulatory relationships into four types: (i) upregulation, which indicates that aging factors upregulate the expression of target genes; (ii) downregulation, which indicates that aging factors downregulate the expression of target genes; (iii) activation, which indicates that aging factors activate the activity of target genes and (iv) inhibition, which indicates that aging factors inhibit the activation of target molecule activity, leading to the declined or lost target activity. It is noteworthy that the changes in the activity and expression of target genes in the aging process cannot be separated from epigenetic modification, so we set up some subcategories of modifications on the basis of regulatory relationships, including ‘binding to promoter’, ‘phosphorylation’, ‘dimerization’, ‘acetylation’, ‘ubiquitylation’ and ‘unclear’. For example, SIRT1 could inhibit P53 acetylation, which induced the antagonistic action of promyelocytic leukemia protein-mediated cell senescence (6). Currently, by perusing thousands of documents, the recent release of AgingReG documents 1345 aging gene entries, 804 external factor entries and eight aging-related pathway entries, involving 651 upregulating pairs, 632 downregulating pairs, 330 activation-regulating pairs and 34 inhibition-regulating pairs, covering 195 disease types and more than 800 kinds of cells and tissue. We expect that AgingReG will help users thoroughly understand the biological processes and in-depth regulatory mechanisms of the aging process. AgingReG is freely available for public use at https://bio.liclab.net/Aging-ReG/.

## Materials and methods

### Data preparation

#### Literature search and review

To ensure high quality of the database, all entries for AgingReG were collected manually as follows: we used the well-recognized hallmarks of aging as keywords for searching in PubMed, such as telomere attrition, mitochondrial dysfunction, epigenetic alterations, loss of proteostasis and cellular senescence. We found nearly 60 000 publications with these hallmarks recorded in the titles or abstracts. Then, we carefully read all the abstracts and retained >20 000 articles that included aging factors in humans. A total of 1784 publications were systematically reviewed finally. We manually extracted the relevant information about aging factors and regulatory relationships validated by experiments. The information on aging factors was divided into three categories: aging genes, external factors and aging-related pathways. Aging-related pathways refer to the change in this pathway itself, which can lead to the acceleration or delay of the aging process.

#### Inclusion criteria

We mainly screened the literature according to two criteria: (i) the authors should verify certain factors that indeed influence the process of aging by performing biological aging–related experiments (e.g. senescence-associated β-galactosidase (SA-β-gal) activity assay, BrdU assay, flow cytometry and Western blot), rather than just prediction. For example, β-gal activity assay was used to detect senescent cells, the cell cycle arrest was verified by flow cytometry and Western blot could suggest the protein changes. (ii) In each literature, it should be clearly pointed out whether aging factors directly affect aging or play a role by regulating target genes.

#### Data annotations

**Figure 1.**
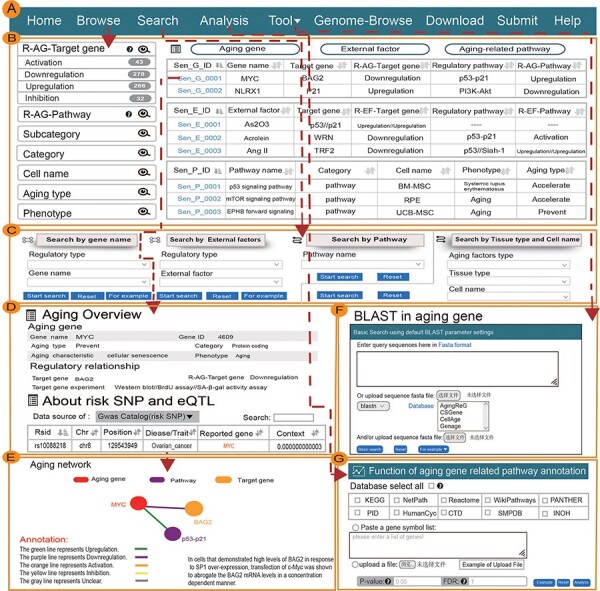
Main function and uses of AgingReG. (A) The top navigation bar helps users use the functions of AgingReG. (B) The snapshot showing the AgingReG browser. (C) Four paths enable searching of specific aging factors and related regulatory relationships. (D) The overview of the details of an aging gene. (E) Target genes potentially associated with aging genes are shown on the basis of five regulatory strategies. Network diagram showing gene–target relationships. (F) BLAST sequences of aging-related genes. (G) Functional annotation of the aging pathway of input data by the hypergeometric test.

We provide two categories of the detailed information on aging factors: basic information and regulatory information. Figure 1 shows the main functions and uses of AgingReg.

‘Basic information’ includes gene name, gene ID, category (protein coding or ncRNA), aging type (accelerate or prevent), aging characteristic (the hallmarks of aging), cell name, tissue type, experimental technology (such as SA-β-gal activity assay, Western blot, knockdown and luciferase report assay), experiment category (low-throughput or high-throughput) and the experimental descriptions recorded in the literature. AgingReG also includes genetic and epigenetic annotations for each aging gene, including common risk single-nucleotide polymorphisms (SNPs) and expression quantitative trait loci (eQTLs). In addition, through the Gene (http://www.ncbi.nlm.nih.gov/gene) and Ensembl databases (http://ensemblgenomes.org/), Human Cell Atlas (7) and CELLPEDIA (8), we standardized the names of genes, tissues and cells.

‘Regulatory information’ includes target genes and regulatory pathways, whose expression is regulated and changed during aging. In the regulatory relationship category, the regulatory information of aging factors includes information on the targets’ name, regulatory type (upregulation, downregulation, activation, inhibition and unclear), experiments and description. The relationships between aging genes and their regulatory objects were named ‘relationships-aging gene (R-AG)-Target gene’ (relationships between aging gene and target gene) and ‘R-AG-Pathway’ (relationships between aging gene and the pathway).

After the completion of the collection process, we obtained 2157 aging factor entries (1345 aging gene entries, 804 external factor entries and eight aging-related pathway entries) and related regulatory information, involving 651 upregulating pairs, 632 downregulating pairs, 330 activation-regulating pairs and 34 inhibition-regulating pairs.

#### Risk SNPs

We downloaded the risk SNPs and genome-wide association studies (GWAS) from GWASdb (9) v2.0 and GWAS catalog (10). Then, we mapped aging genes to risk SNP-associated genes from GWAS to obtain aging gene–related risk SNPs.

#### eQTL

The human eQTL datasets were obtained from PancanQTL (11), HaploReg (12) and GTEx (13) v5.0. Aging gene–related eQTL was considered by mapping aging genes to eQTL genes from the aforementioned three available resources.

#### Aging gene–related pathway analysis

AgingReG provides researchers with a comprehensive functional annotation of the aging pathway. We took the following steps to perform functional prediction: (i) collect comprehensive data about aging genes. We obtained a total of 1345 aging genes with experimental evidence from AgingReG, CSGene (3), CellAge and Genage (5). (ii) Collect the information of 2881 pathways from Kyoto encyclopedia of genes and genomes (KEGG) (14), Reactome (15), NetPath (16), WikiPathways (17), protein analysis through evolutionary relationships (PANTHER) (18), pathway interaction database (PID) (19), HumanCyc (20), comparative toxicogenomics database (CTD) (21), small molecule pathway database (SMPDB) (22) v2,0 and integrating network objects with hierarchies (INOH) (23). (iii) Functional annotation of aging gene–related pathways. A certain pathway contained at least one aging gene, which was considered to be the aging gene–related pathway. Finally, we retained 2293 pathways related to aging genes. Furthermore, AgingReG will calculate the submitted genes with each aging pathway to perform the hypergeometric test. The formula is as follows:


$$P = 1 - \sum\limits_{i = 0}^{K - 1} {\frac{{\left( {_{\mathrm{i}}^M} \right)\left( {_{n - i}^{N - M}} \right)}}{{\left( {_n^N} \right)}}} $$

The *P* value represents the enrichment significance of this aging gene–related pathway. For each pathway, assuming that there are *N* genes in the entire genome, the set of genes of interest has a total of *n* genes, of which *K* genes are involved in the pathway including *M* genes. In addition, the false discovery rate is calculated to correct for multiple testing.

#### Database implementation

In the recent version of AgingReG, the graphical visualization box is based on ECharts and Highcharts and it was built using JBrowse as the Genome browser framework. The design and construction of the interactive interface were based on Bootstrap v3.3.7 and JQuery v2.1.1. The workflow of the database was developed using MySQL 5.7.17, and the server-side script was PHP 7.0 running on a Linux-based Apache Web server. To obtain the best display effect, we recommend using a modern web browser that supports the HyperText Markup Language 5 standard, such as Firefox, Google Chrome, Safari, Opera or ie9.0 + . In addition, for ease of use, users can access the database without registering or logging in.

## Results

### Database content

**Figure 2.**
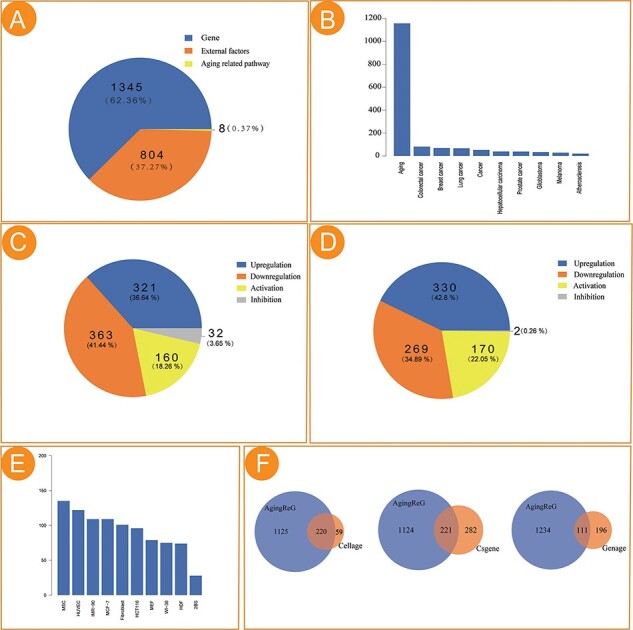
Statistical analysis of the aging factors in AgingReG. (A) The number of aging factors in AgingReG. (B) The distribution of the pathological phenotypes associated with aging factors in AgingReG. The top 10 extensively studied pathological phenotypes recorded in AgingReG. (C) The number of aging gene–related regulatory relationship entries. (D) The number of external factor–related regulatory relationship entries. (E) The top 10 frequently studied cells in aging research in AgingReG. (F) The comparison between AgingReG and Cellage, between AgingReG and CSGene and between AgingReG and GenAge and the number of overlapping data points.

The current version of AgingReG integrates 1345 aging-related gene entries, 804 external factor entries and eight aging-related pathway entries. The number of genes, external factors (e.g. radiation, gas and chemical compounds) and pathways accounting for 62.36%, 37.27% and 0.37%, respectively, are included (Figure 2A). Of all the entries, a total of 997 entries were identified on the basis of their relationships with lung cancer, colorectal cancer, breast cancer and other disease phenotypes (Figure 2B). In cardiovascular disease, the signal transducer and activator of transcription 3 (STAT3) pathway regulated the expression of p21 and ICAM-1 and cryptotanshinone inhibition of STAT3 prevented H19 consumption–mediated p21 induction and delayed aging (24). Western blot analysis confirmed that the level of endogenous SIRT1 protein was severely reduced by overexpression of MIR34A, while the level of exogenous SIRT1 protein was not affected, which delayed the aging process in cardiovascular diseases (25). Among the regulatory relationships between aging genes and target genes, 321 pairs of relationships (36.64%) were ‘Upregulation’, 363 pairs of relationships (41.44%) were ‘Downregulation’, 160 pairs of relationships (18.26%) were ‘Activation’ and 32 pairs of relationships (3.65%) were ‘Inhibition’ (Figure 2C). Similarly, the statistics of the regulatory relationships between external factors and target genes are also provided (Figure 2D). The most frequently studied cells in aging research in AgingReG were also shown (Figure 2E). We explored the overlap between AgingReG and databases previously published to be related to aging; there are 220 genes that overlapped with CellAge, 221 genes that overlapped with CSGene and 111 genes that overlapped with Geneage. Approximately 1110 genes were specifically collected in the AgingReG platform, compared with 59, 282 and 196 genes specifically collected in the CellAge, CSGene and Geneage databases, respectively (Figure 2F). Along with other databases, such as DrugAge and AgeFactDB, focusing on the effects of drugs, compounds or other factors on longevity on the basis of predictive and analytical data, AgingReG focuses on the molecular regulatory mechanisms involved in the aging process, including the specific regulatory relationship between aging factors and target genes and providing life scientists with more systematic integration data and records.

### User interface

The AgingReG database provides a convenient aging factor data retrieval interface on the Search page. Users can search in four paths to obtain aging factors and related regulatory information, such as ‘Search by gene name’, ‘Search by external factors’, ‘Search by pathway’ and ‘Search by tissue type and cell name’. A summary of the search results was displayed in a table on the results page. Users can click ‘More details’ to view detailed information about each aging factor, such as the relationship between aging genes, including basal gene annotation (‘Aging gene name’, ‘Target gene’, ‘Gene ID’ and ‘Category’), literature records (‘Phenotype’, ‘Aging type’, ‘Aging characteristic’, ‘Tissue type’ and ‘Experiment category’) and descriptions of experiments in the literature. It is worth noting that when an aging gene regulates several target genes, we use the symbol ‘//’ to distinguish and correspond to each regulatory relationship. In addition, the network is displayed visually, showing the relationships between aging genes and their regulatory information, with different colored lines representing regulatory relationships (for example, upregulation, downregulation, activation, inhibition and unclear). Based on the regulatory relationships, we read and extracted the modifications in publications and classified them into ‘binding to promoter’, ‘phosphorylation’, ‘dimerization’, ‘acetylation’, ‘ubiquitylation’ and ‘unclear’. In addition, users can obtain regulatory information about experimental aging factors by searching external factors, pathways, tissue types and cell names. AgingReG allows users to download required data and submit new data through the download and submission pages, providing users with detailed steps to follow.

The AgingReG database provides a user-friendly ‘Data browsing’ page, which is organized into an interactive alphanumeric sorting table, allowing users to quickly browse ‘Gene name’, ‘Gene ID’, ‘Target gene’, ‘Regulatory type’, ‘Phenotype’ and ‘Aging type’. Users can quickly browse the regulatory relationships, aging genes and related information of interest through the fuzzy search function and easily change the number of records per page through the ‘Display item’ drop-down menu. We coded and stored the aging factors. Users can click on ‘Sen_G_ID’ to view detailed information about aging factors and related genes of interest. In addition, on the details page, the regulatory relationships of each aging factor with downstream molecules are visualized and each regulatory pathway is divided into two types: one is the official pathway whose name is relatively clear because it directly matches the official name of the pathway, and the other type shows the regulatory pathway after adjusting to a gene set and displays all the related pathways of the standardized regulatory pathways. Additionally, AgingReG also displays the expression levels of aging genes and target genes in different tissues and cells, and these gene expression data were integrated with GTEx (26), cancer cell line encyclopedia (https://sites.broadinstitute.org/ccle/) and encyclopedia of DNA elements (27).

The AgingReG database provides a personalized genome browser and intuitive data visualization. To help users view the proximity of aging genes and their mediation annotation in the genome, we developed a personalized genome browser using JBrowse and added many useful tracks, such as aging genes and related risk SNPs. In addition, the roles played by aging genes in different studies and the types of cells analyzed can be simultaneously displayed.

To help users identify unknown aging-related genes, we performed a multigene sequence comparison. The AgingReG database provided a practical tool for comparing gene sequences. The sequences of each gene of interest were aligned against the other experimentally confirmed aging-related genes by BLAST, and the similarity of the sequences was obtained and provided for users’ reference (28).

In AgingReG, the user can manually input a set of genes of interest (one gene symbol per line) or upload the gene set in a text file and then select at least one pathway database (containing KEGG, Reactome, NetPath, WikiPathways, PANTHER, PID, HumanCyc, CTD, SMPDB and INOH) to perform the function of aging pathway annotation. Users can also modify the number of genes inputted and set the *P* value to control the stringency of the analysis. In addition to online analysis, AgingReG also provided convenient data downloads, including both aging genes data and information about aging pathways.

## Discussion

By integrating the experimental regulatory interactions among genes, external factors and pathways from thousands of literature, we developed a database named AgingReG that serves as a comprehensive repository of regulatory relationships in human aging. In the field of aging research, Aging Atlas integrates aging-related multi-omics datasets and CSGene and GenAge have been constructed for various genetic studies on cell senescence. In contrast to these databases, AgingReG focuses on the complex regulatory relationships in aging with experimental evidence. GenAge contains a number of genes related to aging and presents a protein network of human aging, but the regulatory relationships between gene and protein confirmed by Western blot, pull-down, luciferase reporter assays and other low-throughput experiments were not included. AgingReG provides this information and further classifies the regulatory relationships into four types. In addition, we also mapped aging genes with risk SNPs and eQTL, which will help users deepen their understanding of the genetic information of aging genes.

Besides containing large quantities of aging gene entries, AgingReG presents several features that distinguish it from previously developed aging databases: (i) AgingReG focuses on the regulatory relationships between aging factors and target genes and classifies the specific types of regulation into upregulation, downregulation, activation regulation and inhibition regulation. (ii) AgingReG also collates and refines the gene regulatory relationships of external factors during aging, which is helpful for researchers to quickly understand the regulatory mechanism of compounds or other external factors in aging. (iii) AgingReG is committed to collecting functional and annotated information on aging factors and regulatory relationships that are supported by experimental evidence. The aforementioned regulatory relationships were supported by experimental evidence, such as Western blot, co-immunoprecipitation, pull-down and other low-throughput biological experiments. (iv) With the help of two online analytical tools of AgingReG, users can predict the potential aging genes and obtain aging gene–related pathway functional annotation. (v) AgingReG provides users with the expression levels of aging genes and target genes in different tissues and cells, which can also be visualized on the details page. (vi) AgingReG integrates the information of risk SNPs related to aging genes so that users can quickly understand the genetic information related to aging genes while searching for them, which is convenient for user analysis of the biological processes of aging in an integrative and system-level way. (vii) AgingReG is based on a stricter and more detailed selective procedure. By searching the hallmarks of aging as keywords, AgingReG retrieves the most comprehensive aging-related articles to the greatest possible extent and extracts information according to rigorous standards. (viii) AgingReG also provides information on relevant established experiments with cells and tissue, including experimental methods and experimental descriptions that users can access to make experimental hypotheses.

In summary, AgingReG is an easy-to-use database platform with which researchers can further broaden the molecular picture of aging and associated genetic mechanisms. We will continuously update and replenish AgingReG. In the future, we plan to add more regulatory relationships related to aging and expand the number of model organisms with aging genes, longevity genes, inflammation-related genes and the related regulatory information. We believe that AgingReG will become a valuable resource database in the field of aging research and provide a more convenient and practical platform with the aim of finding new anti-aging and anti-tumor treatment methods.

## Data Availability

The AgingReG database is freely available to all users without the requirement to register or login. These data can be found at https://bio.liclab.net/Aging-ReG/.
